# Uncovering the Novel Role of NR1D1 in Regulating BNIP3-Mediated Mitophagy in Ulcerative Colitis

**DOI:** 10.3390/ijms241814222

**Published:** 2023-09-18

**Authors:** Yidong Chen, Junrong Li, Shuang Li, Yiyu Cheng, Xiaoyu Fu, Jiamin Li, Liangru Zhu

**Affiliations:** Division of Gastroenterology, Union Hospital, Tongji Medical College, Huazhong University of Science and Technology, Wuhan 430022, China

**Keywords:** circadian clock, ulcerative colitis, NR1D1, BNIP3, mitophagy

## Abstract

Background: Ulcerative colitis (UC) is a chronic, incurable condition characterized by mucosal inflammation and intestinal epithelial cell (IEC) damage. The circadian clock gene NR1D1, implicated in UC and the critical mitophagy process for epithelial repair, needs further exploration regarding its role in mitophagy regulation in UC. Methods: We created a jet lag mouse model and induced colitis with dextran sulfate sodium (DSS), investigating NR1D1’s role. Intestinal-specific Nr1d1 knockout mice were also generated. RNA sequencing, chromatin immunoprecipitation (ChIP), and dual-luciferase reporter assays helped ascertain NR1D1’s regulatory effect on BNIP3 expression. The mitochondrial state in IECs was assessed through transmission electron microscopy, while confocal microscopy evaluated mitophagy-associated protein expression in colon tissue and CCD841 cells. Cell apoptosis and reactive oxygen species (ROS) were measured via flow cytometry. Results: We observed reduced NR1D1 expression in the IECs of UC patients, accentuated under jet lag and DSS exposure in mice. NR1D1 ablation led to disrupted immune homeostasis and declined mitophagy in IECs. NR1D1, usually a transcriptional repressor, was a positive regulator of BNIP3 expression, leading to impaired mitophagy, cellular inflammation, and apoptosis. Administering the NR1D1 agonist SR9009 ameliorated colitis symptoms, primarily by rectifying defective mitophagy. Conclusions: Our results suggest that NR1D1 bridges the circadian clock and UC, controlling BNIP3-mediated mitophagy and representing a potential therapeutic target. Its agonist, SR9009, shows promise in UC symptom alleviation.

## 1. Introduction

Inflammatory bowel disease (IBD), which comprises Crohn’s disease (CD) and ulcerative colitis (UC), is a chronic, recurrent, and currently incurable condition. CD is characterized by patchy lesions in the gastrointestinal tract and transmural inflammation, leading to fistula, stricture, and fibrosis [1]. UC is growing in prevalence and is characterized by weight loss, diarrhea, rectal bleeding, abdominal pain, and mucosal inflammation that extends from the rectum to the distal colon [2]. Despite considerable research, a complete understanding of UC’s pathophysiology remains elusive. However, the disturbance and damage of the colonic mucosa have been implicated, facilitating the intrusion of microorganisms and antigens into the gut wall and instigating an unchecked immune response [3,4]. 

The colonic mucosa comprises intestinal epithelial cells (IECs) and an inner mucus layer [5]. IECs maintain intestinal homeostasis by secreting mucins and antimicrobial peptides, coordinating immune responses against commensal and pathogenic microorganisms, and delivering luminal bacteria and antigens to antigen-presenting colonic lamina propria cells [6,7]. Furthermore, mesenchymal and epithelial cells, along with the microbial microenvironment, determine immune cell function, diversity, and compartmentalization throughout the intestinal tract [8]. This highlights the critical role that IECs play in maintaining intestinal homeostasis.

IECs are characterized by an impressively rapid renewal cycle, refreshing every 3 to 5 days [9]. This process is critically dependent on robust mitochondrial metabolism and functionality [10]. Mitochondria govern energy production, metabolism, cellular homeostasis, and apoptosis [11,12]. Moreover, mitochondria serve as signal transduction hubs that regulate cellular innate and adaptive immunity [13,14]. Although it remains uncertain whether mitochondrial damage is the progenitor or the product of IBD, there is a growing body of evidence suggesting that mitochondrial dysfunction, through the modulation of cell death, plays a significant role in IBD pathogenesis [12,15]. In this respect, the dysregulation of intestinal epithelial cell apoptosis is associated with IBD pathogenesis and severity [16]. 

Following mitochondrial damage, cells rapidly repair or remove damaged mitochondria to prevent cell death or tissue damage. One of the key pathways for mitochondrial quality control is mitophagy, a highly specialized form of autophagy dedicated to the removal of dysfunctional or damaged mitochondria [17]. Mitophagy, a selective form of autophagy, is mediated by both PRKN-dependent and PRKN-independent pathways. PRKN, also known as Parkin, is an E3 ubiquitin ligase that plays a pivotal role in the mitophagic process. Upon mitochondrial depolarization, PRKN is recruited to the mitochondria, where it ubiquitinates outer mitochondrial membrane proteins, marking them for selective degradation by the autophagolysosome [18]. BNIP3 (BCL2/adenovirus E1B 19 kDa protein-interacting protein 3) is another protein that mediates mitophagy, albeit through a PRKN-independent mechanism. BNIP3 is a BH3-only protein that can induce mitochondrial depolarization. It targets mitochondria and triggers the autophagolysosome pathway for mitochondrial degradation, thereby playing a crucial role in mitochondrial quality control [19]. These processes are of particular relevance to UC [20], as mitophagy has been shown to regulate various cellular functions, including the production of inflammatory factors [21], reactive oxygen species, and the overall survival of IECs [22].

The endogenous circadian clock harmonizes molecular and physiological processes with the diurnal light–dark cycle [23]. In mammals, the suprachiasmatic nucleus, the central pacemaker, synchronizes endogenous rhythms with the light–dark cycle [24]. Circadian rhythms are orchestrated through a complex interplay of transcriptional and translational negative feedback loops that encompass a range of clock genes and associated proteins. The *CLOCK* (Circadian Locomotor Output Cycles Kaput) and *BMAL1* (Brain and Muscle Arnt-Like 1) genes serve pivotal roles in driving this system as positive regulators. Concurrently, Period (*PER*), Cryptochrome (*CRY*), and *NR1D1* (Nuclear Receptor Subfamily 1, Group D, Member 1) genes act to impede the function of the BMAL1-CLOCK heterodimer [25], thereby embodying the negative regulatory facet of this intricate temporal cycle. As a member of the 1D transcription factor family, NR1D1 exerts a broad influence over a spectrum of cellular processes. Its functional breadth encompasses the regulation of cell proliferation and differentiation, modulation of mitochondrial energy production, steering of innate immune responses, and oversight of autophagic pathways [26,27,28]. Emerging evidence suggests that circadian rhythm disruptions, such as those experienced in jet lag, may also play a significant role in the etiology and exacerbation of IBD, particularly UC [29,30,31]; however, the relationship between specific circadian clock genes and UC remains unclear. 

In the landscape of IBD, UC presents a complex pathophysiology that demands thorough exploration for effective therapeutic intervention. Among various molecular candidates, we focused on the NR1D1 gene for this study. This gene has been observed to have reduced expression in both UC and conditions like jet lag. Given that jet lag has been shown to compromise the immune homeostasis of IECs, investigating NR1D1 holds potential for revealing new dimensions in the pathophysiology of UC, particularly in relation to epithelial dysfunction. To delve into this, we established jet-lag and UC mouse models and discovered that NR1D1 plays a pivotal role in modulating UC within IECs. Knockout experiments demonstrated that the deletion of NR1D1 reduced BNIP3 expression, thereby impairing mitophagy and exacerbating UC symptoms. Moreover, NR1D1 knockdown was found to amplify TNFα-induced mitophagy, escalating cell apoptosis. These findings lay a robust foundation for uncovering the pathogenesis of UC and identifying novel therapeutic targets.

## 2. Results

### 2.1. Circadian Clock Gene Expression Patterns in IECs Altered by Jet Lag and Ulcerative Colitis

**Figure 1:**
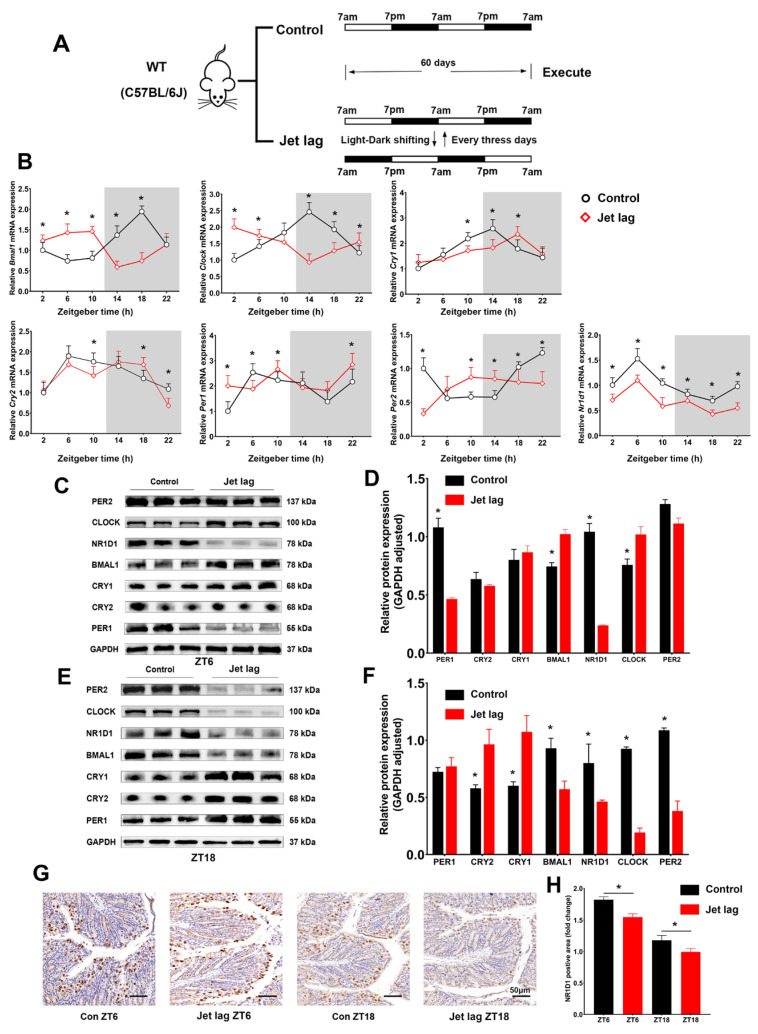
Jet lag alters clock gene expression in intestinal epithelial cells (IECs). (**A**) Schematic of the jet lag mouse model. (**B**) Quantitative real-time PCR (qPCR) analysis of the relative mRNA expression of core clock genes in IECs from jet lag and control mice, normalized to GAPDH mRNA levels. (**C**–**F**) Western blot assays and semi-quantitative densitometric evaluations of core clock gene protein expression in IECs at zeitgeber times 6 and 18. (**G**) Immunohistochemical examination of NR1D1 expression at zeitgeber times 6 and 18, with a scale bar representing 50 μm. (**H**) Quantification of NR1D1-positive staining areas. Data are represented as means ± standard deviations (*n* = 3–6). * denotes *p* < 0.05 (Student’s *t*-test).

In an endeavor to ascertain the role of circadian clock genes in the IECs, we instituted a jet-lag mouse model. This was achieved by inducing alterations in the light–dark cycle, wherein lights were switched on at 7 a.m. and off at 7 p.m. daily, interspersed with a 12 h phase shift every 3 days (Figure 1A). By utilizing qPCR, we examined the relative expression of core circadian clock genes in IECs at six time points. In the jet lag model, we observed a significant shift in the temporal expression patterns of most genes (Figure 1B). Western blot analysis and immunohistochemistry further confirmed these findings, albeit only at two representative time points -ZT 6 and ZT18 (Figure 1C–H). Among these, a striking observation was the consistent decrease in NR1D1 expression across all examined time points.

**Figure 2:**
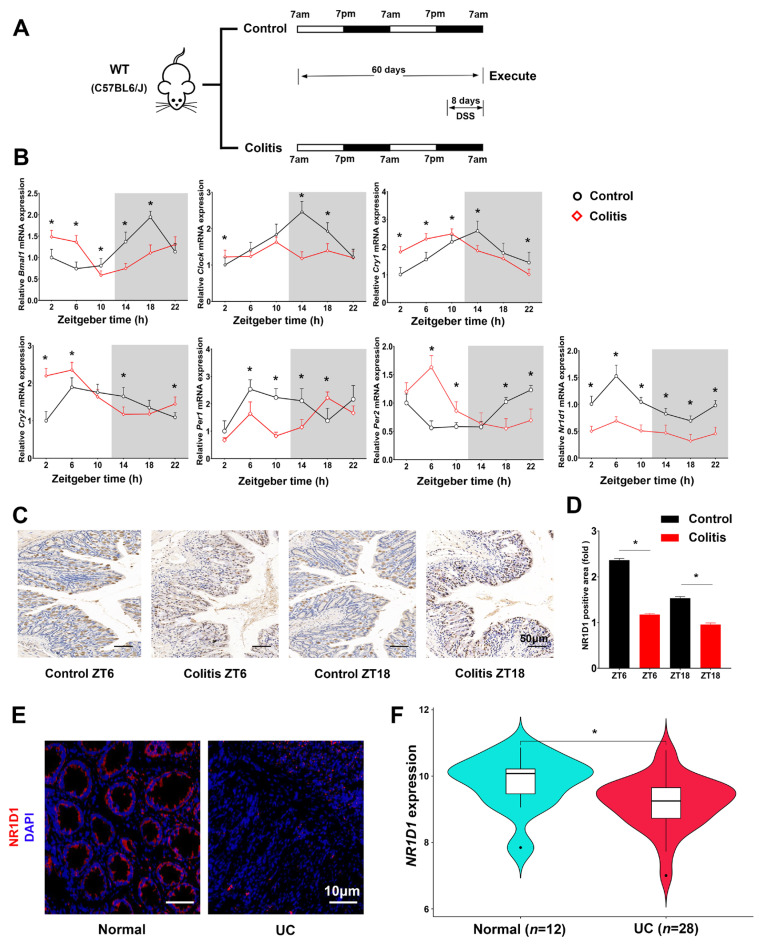
Colitis modifies circadian gene expression in IECs. (**A**) Schematic depiction of the colitis mouse model. (**B**) qPCR analysis of the relative mRNA expression of core clock genes in IECs from colitis-induced and control mice, normalized to GAPDH mRNA levels. (**C**) Immunohistochemical investigation of NR1D1 expression in IECs at zeitgeber times 6 and 18, scale bar = 50 μm. (**D**) Quantitative assessment of NR1D1-positive staining regions. (**E**) Immunofluorescence-based evaluation of NR1D1 expression in colonic biopsy specimens from UC patients and control subjects, with a scale bar representing 10 μm. (**F**) Comparative expression of NR1D1 between UC patients and non-UC subjects utilizing the GSE53306 database. Data are represented as means ± standard deviations (*n* = 3–6). * denotes *p* < 0.05 (Student’s *t*-test).

Colitis was induced in mice using DSS, simulating the conditions observed in human inflammatory bowel diseases, inclusive of ulcerative colitis [32] (Figure 2A). Compared to the control group (CG), colitis altered most genes’ temporal expression patterns (Figure 2B). Notably, NR1D1 expression was also reduced at all six time points. This result was confirmed by immunohistochemistry (Figure 2C,D).

Immunofluorescence showed that NR1D1 expression was low in the colonic biopsies of UC patients compared to healthy controls (Figure 2E). The data on NR1D1 expression in colonic biopsy specimens were retrieved from the NCBI Gene Expression Omnibus database (GSE53306). The results indicated that NR1D1 expression was lower in UC patients than in healthy individuals (Figure 2F). These data confirm that jet lag and UC dysregulate clock gene expression in IECs and decrease NR1D1 expression. Thus, we hypothesize that NR1D1 is a crucial link between the circadian clock and UC.

### 2.2. Downregulation of NR1D1 Impairs Immune Homeostasis in IECs and Exacerbates DSS-Induced Colitis in Mice

**Figure 3:**
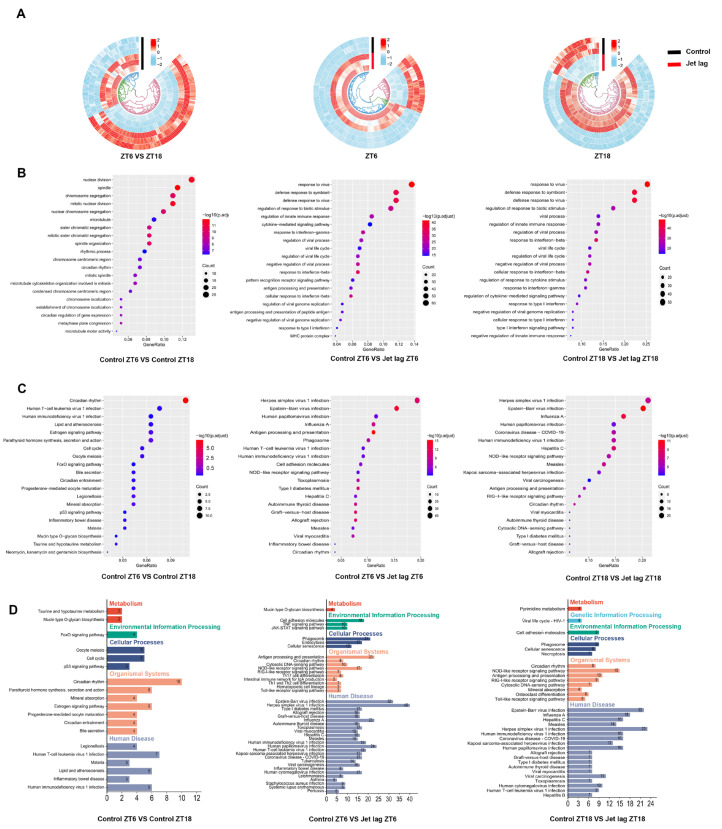
Jet lag disrupts immune homeostasis in IECs. (**A**) Heat maps exhibiting differentially expressed genes (DEGs) between zeitgeber times (ZTs) 6 and 18 within the control group (CG) (left panel) and between the CG and the jet lag group at both ZT6 and ZT18 (right panels). (**B**) Gene Ontology (GO) enrichment analysis depicting DEGs in the CG across ZT6 and ZT18 (left panel) and those between the CG and jet lag group at both ZT6 and ZT18 (right panels). (**C**) Kyoto Encyclopedia of Genes and Genomes (KEGG) pathway enrichment analysis showing DEGs in the CG between ZT6 and ZT18 (left panel) and those between the CG and jet lag group at ZT6 and ZT18 (right panels). (**D**) Classification and distribution of enriched KEGG pathways. Experimental replicates *n* = 3.

To confirm the role of NR1D1 in UC, RNA sequencing was performed in IECs isolated from jet lag and control mice at ZT6 and ZT18. There were DEGs between the experimental and control mice at the two time points (Figure 3A). Gene Ontology and Kyoto Encyclopedia of Genes and Genomes pathway enrichment analyses of the DEGs revealed significant alterations in IECs induced by jet lag. Relative to the CG, the jet lag group demonstrated an enrichment of biological processes related to immune response and cytokine activity. Furthermore, various pathways tied to infection and immune function were prominently implicated in the jet lag group (Figure 3B,C). DEGs were further classified into different putative functional groups. The results showed that several DEGs in the jet lag group were enriched in pathways associated with human diseases (Figure 3D). Considering that in the KEGG enrichment analysis of Control ZT6 and ZT18, only circadian rhythm had a statistical difference, these results suggest that jet lag impairs immune homeostasis in IECs.

**Figure 4:**
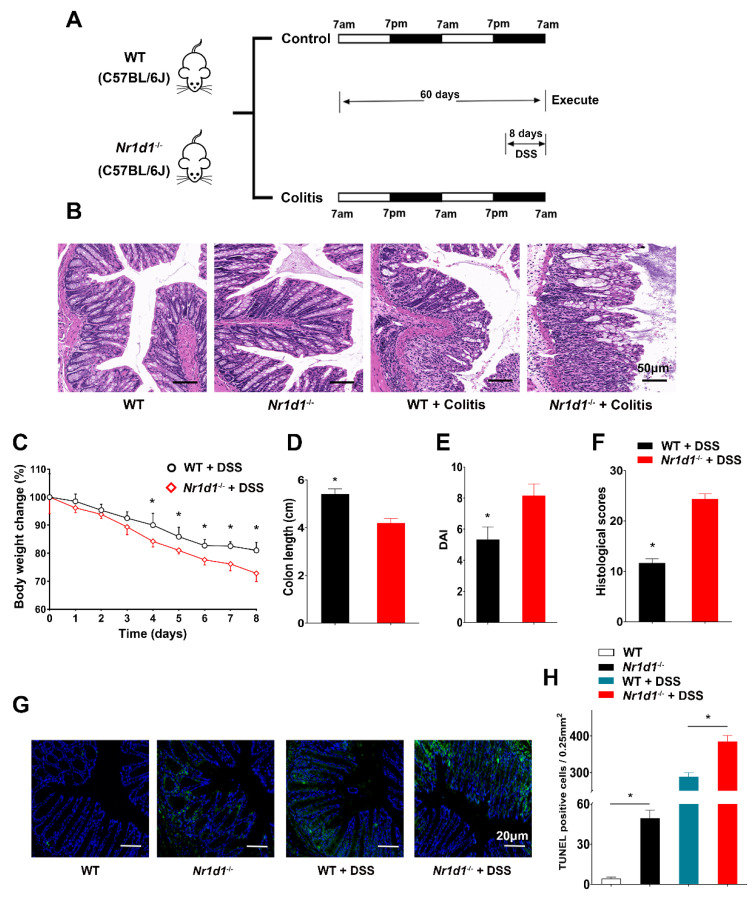
*Nr1d1* deletion intensifies colitis severity. (**A**) Schematic representation of the colitis mouse model. (**B**) Hematoxylin and eosin staining of the colon from wild-type and *Nr1d1*^−/−^ mice subjected to colitis, scale bar = 50 μm. (**C**) Body weight dynamics of the groups. (**D**–**F**) Comparative assessments of colon length, disease activity index scores, and histological scores between the experimental cohorts. (**G**,**H**) Quantitative analysis of TUNEL-positive cells in colonic samples derived from wild-type and *Nr1d1*^−/−^ mice, with and without colitis induction, scale bar = 20 μm. Data points are expressed as means ± standard deviations (*n* = 3–6). * *p* < 0.05 (Student’s *t*-test).

Colitis was induced in WT and Nr1d1^−/−^ mice using DSS (Figure 4A). Colitis was more severe in Nr1d1^−/−^ mice than in WT mice, with significant damage to the colonic mucosa (Figure 4B). Furthermore, Nr1d1^−/−^ mice had higher weight loss, shorter colon length, and higher histopathological and disease activity index (DAI) scores than the CG (Figure 4C,F). Considering that intestinal epithelial apoptosis is dysregulated in UC patients [33,34], we measured apoptosis in the IECs of mice through TUNEL staining. The results demonstrated that Nr1d1 KO increased the number of TUNEL-positive cells, and DSS enhanced this effect (Figure 4G,H). To further substantiate these findings, we conducted an ELISA assay to measure the expression levels of TNFα, IL-6, IL-10, and IL-β in IECs (Figure S1). Remarkably, the absence of Nr1d1 led to an increase in the expression levels of TNFα, IL-6, and IL-β and a decrease in IL-10 under DSS-induced conditions. These data confirm that NR1D1 plays an essential role in the homeostasis of IECs and regulates the circadian clock and UC.

### 2.3. NR1D1 Has Been Identified as a Transcription Factor That Regulates the Expression of BNIP3

**Figure 5:**
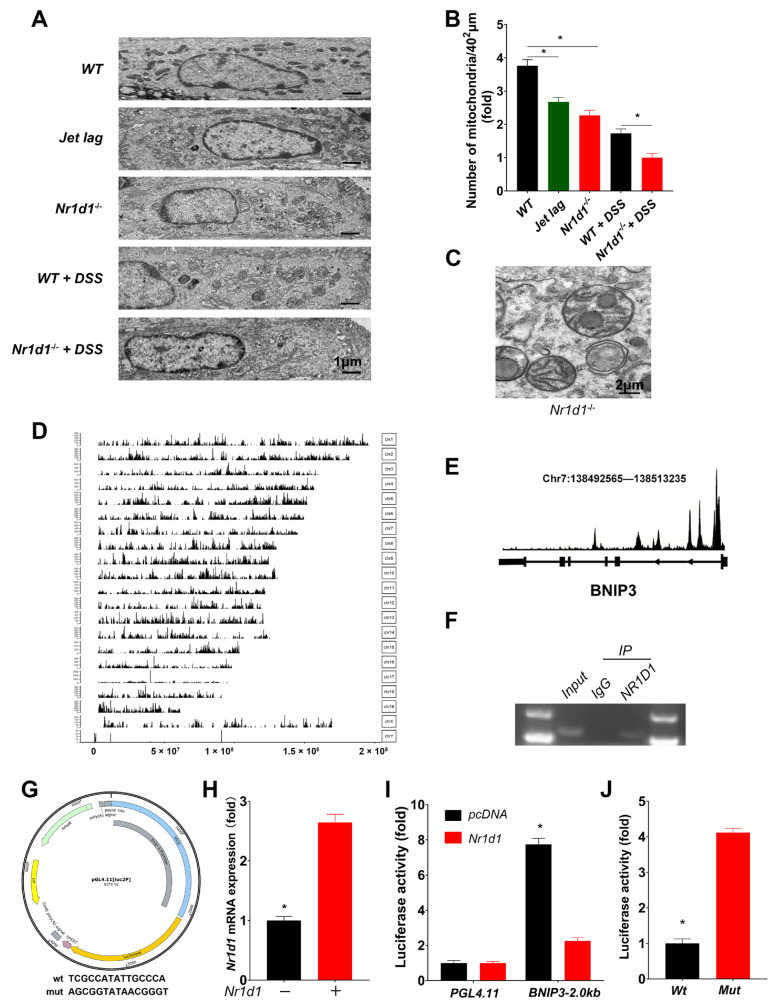
NR1D1 promotes BNIP3 transcription. (**A**) Representative transmission electron microscopy (TEM) images showcasing mitochondrial structures in IECs derived from wild-type (WT) and *Nr1d1*^−/−^ mice from a jet lag model, as well as WT and *Nr1d1*^−/−^ mice treated with DSS, with a scale bar representing 1 μm. (**B**) Quantification of damaged mitochondria per field of view. (**C**) Representative image of mitochondrial diameter, scale bar = 2 μm. (**D**) ChIP-seq signal peak distributions across chromosomes using an anti-NR1D1 antibody, with the X-axis signifying chromosomal length and the Y-axis indicating signal peak frequency along the chromosome. (**E**) BNIP3 locus depiction using the Peak Browser tool. (**F**) Agarose gel electrophoresis of ChIP-PCR products. (**G**) Schematic representation of the reporter plasmid (mut: mutation in the BNIP3 promoter region at the NR1D1 binding site). (**H**) Quantitative real-time PCR analysis of *Nr1d1* mRNA expression in CCD 841 CoN cells post *Nr1d1* overexpression plasmid transfection. (**I**,**J**) Evaluation of luciferase activity in CCD 841 CoN cells using dual-luciferase reporter assays. Data are displayed as means ± standard deviations (*n* = 3). * *p* < 0.05 (Student’s *t*-test and one-way analysis of variance).

Since mitochondrial function is crucial for cellular homeostasis [10,35], we utilized transmission electron microscopy to visualize mitochondria in IECs. Nr1d1 KO leads to a decrease in mitophagy, causing an accumulation of damaged mitochondria in IECs under inflammatory conditions. This process triggers a morphological transformation of the mitochondria, with them becoming swollen and rounded, accompanied by a reduction in their diameter (Figure 5A–C). Given that NR1D1 is a transcription factor that governs the transcriptional activities of several genes, we proposed that Nr1d1 KO could impact the expression of a particular gene pivotal to mitophagy, resulting in impaired mitophagy. To validate this hypothesis, we delved into the NR1D1 ChIP-seq data (GSE104129) (Figure 5D). An examination of the ChIP read pileup alongside the detected BNIP3 peaks revealed a significant concentration of reads at the transcription start site (TSS) (Figure 5E), suggesting a plausible role of NR1D1 as a transcriptional regulator of BNIP3. Prompted by these findings, we proceeded to conduct ChIP and dual-luciferase validation assays in CCD841 CoN cells. The results from the ChIP-PCR analysis corroborated that BNIP3 was indeed enriched by the anti-NR1D1 antibody (Figure 5F). Dual-luciferase reporter assays revealed augmented luciferase activity from the reporter gene harboring the wild-type BNIP3 promoter (pGL4.11-WT) upon overexpression of NR1D1 (Figure 5G–I). Conversely, the introduction of a mutation 7 base pairs upstream of the transcription start site within the BNIP3 promoter region of the reporter gene (pGL4.11-mut) abrogated this effect (Figure 5J). 

In order to verify the potential bidirectionality of NR1D1 and BNIP3 regulation, we executed BNIP3 overexpression in CCD841 CoN cells and examined the subsequent impact on NR1D1 expression through Western blot analyses. Remarkably, BNIP3 overexpression did not elicit any detectable changes in the NR1D1 protein levels (Figure S2). This observation lends further credence to the concept that NR1D1 acts as a unidirectional transcriptional regulator of BNIP3, without a reciprocal influence from BNIP3 on NR1D1 expression. These findings indicate that NR1D1 enhances BNIP3 expression, whereas Nr1d1 KO results in compromised mitophagy, implying a critical role for NR1D1 in modulating mitophagy under inflammatory conditions.

### 2.4. Nr1d1 KO Leads to Impaired Mitophagy

**Figure 6:**
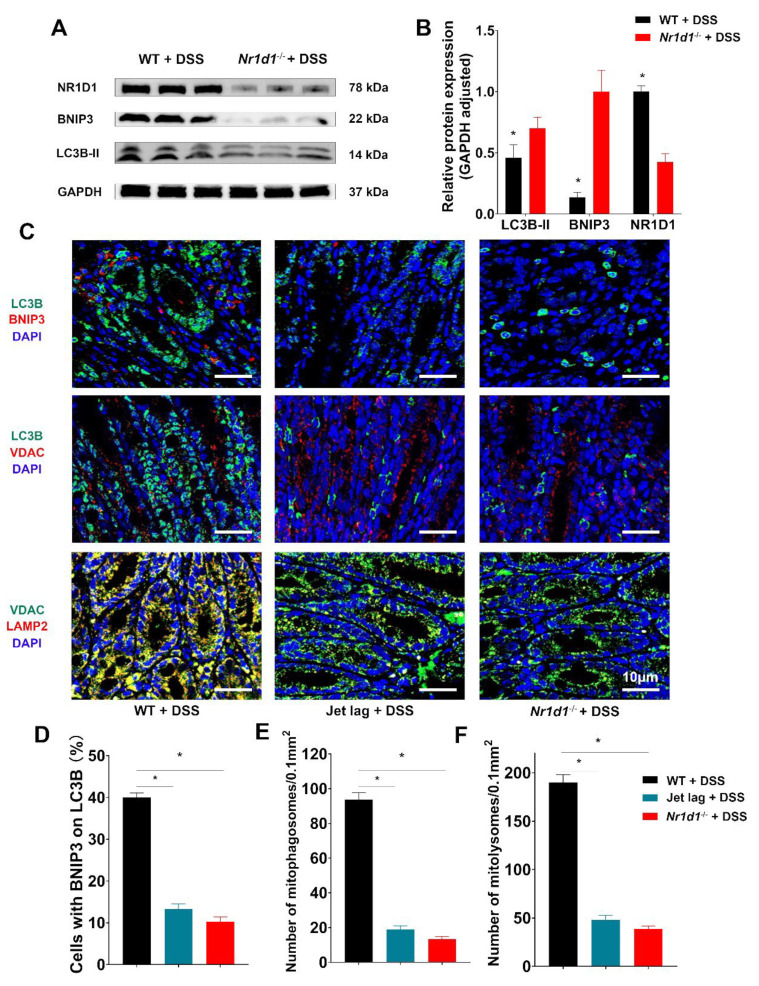
*Nr1d1* knockout impairs mitophagy. (**A**,**B**) Western blot analysis along with semi-quantitative densitometric evaluations of NR1D1, BNIP3, and LC3B-II protein expression in mouse intestinal epithelial cells (IECs). (**C**) Immunofluorescence-based analysis of colocalization between LC3B and BNIP3; LC3B and VDAC; and VDAC and LAMP2 within IECs, scale bar = 10 μm. (**D**–**F**) Quantifications of cells concurrently expressing BNIP3 and LC3B, the number of mitophagosomes per 0.1 mm^2^, and the number of mitolysosomes per 0.1 mm^2^. Data are illustrated as means ± standard deviations (*n* = 3). * *p* < 0.05 (Student’s *t*-test and one-way analysis of variance).

To further investigate the influence of NR1D1 depletion on BNIP3 expression and mitophagy, we conducted in vivo experiments with mice. A protein expression analysis revealed that Nr1d1 KO led to a decrease in BNIP3 and LC3B-II expression in IECs (Figure 6A,B). Moreover, immunofluorescence analyses suggested a decrease in the co-localization of LC3B and BNIP3, LC3B and VDAC, as well as VDAC and the lysosomal marker LAMP2 in NR1D1 knockout samples (Figure 6C–F). These findings highlight the significant impact of NR1D1 ablation on BNIP3 expression and mitophagy in IECs.

**Figure 7:**
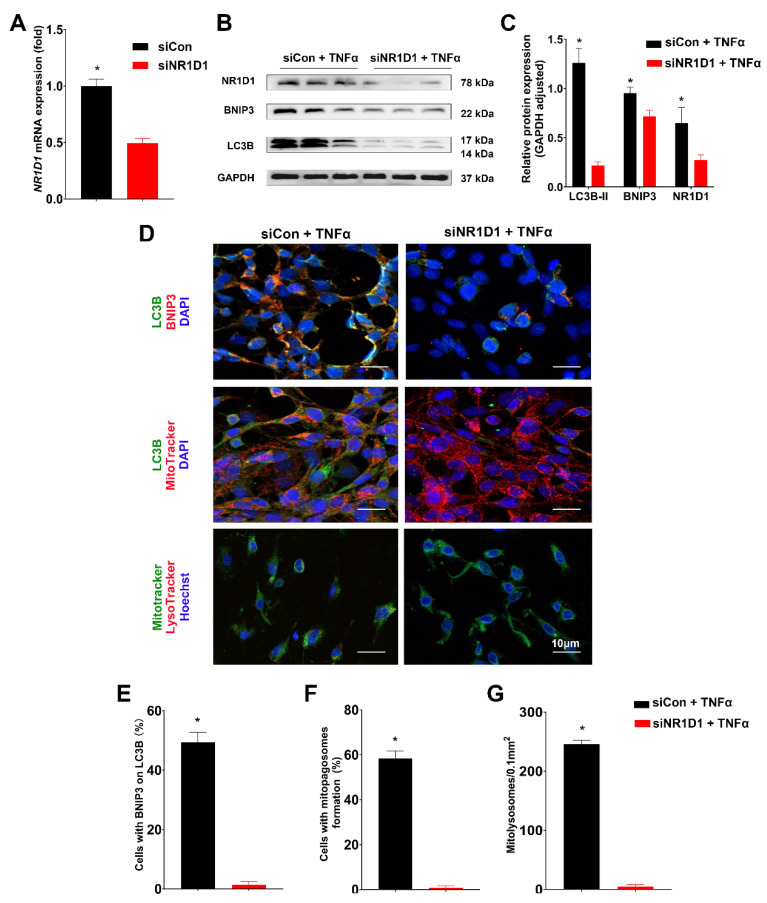
Knockdown of *NR1D1* decreases mitophagy in CCD 841 CoN cells. (**A**) Assessment of relative mRNA expression of NR1D1 in CCD 841 CoN cells post treatment with siNR1D1. (**B**,**C**) Following TNFα stimulation, protein expression of NR1D1, BNIP3, and LC3B-II is evaluated using immunoblotting and analyzed via semi-quantitative densitometric analysis. (**D**) Immunofluorescence microscopy depicting the colocalization of LC3B and BNIP3, LC3B and MitoTracker, and MitoTracker and LysoTracker in TNFα-stimulated cells, scale bar = 10 μm. (**E**–**G**) Quantitative analysis of TNFα-stimulated cells co-expressing BNIP3 and LC3B, as well as enumeration of mitophagosomes and mitolysosomes per 0.1 mm^2^ field of view post-TNFα treatment. Data are presented as means ± standard deviations (*n* = 3–6). * *p* < 0.05 (Student’s *t*-test).

In our in vitro validation phase, CCD841 CoN cells were transfected with either si-NR1D1 or si-Control and subsequently stimulated with TNFα at 50 ng/mL for 12 h. Upon transfection with NR1D1-siRNA, we observed a significant reduction in NR1D1 mRNA expression, as confirmed by the PCR analysis (Figure 7A). Additionally, the corresponding decrease in NR1D1 protein levels was evident in the Western blot analysis provided in Supplementary Figure S3.

Intriguingly, following NR1D1 knockdown (KD) and subsequent TNFα stimulation, we observed a marked decrease in the levels of BNIP3 and LC3B-II proteins (Figure 7B,C). Moreover, this TNFα-induced stimulation in the context of NR1D1 KD resulted in a diminished colocalization of LC3B-II with BNIP3, LC3B-II with MitoTracker, and MitoTracker with LysoTracker, compared to the CG (Figure 7D). These results suggest that TNFα stimulation in NR1D1-depleted cells leads to both enhanced mitochondrial damage and impaired mitophagy. Furthermore, upon NR1D1 KD and TNFα stimulation, we observed a significant reduction in the formation of mitophagosomes and mitolysosomes (Figure 7E–G). Collectively, these findings strongly suggest that depletion of NR1D1, particularly when cells are stimulated with TNFα, impairs mitophagy, potentially due to a decrease in BNIP3 expression.

### 2.5. NR1D1 Modulation by SR9009 Mitigates TNFα-Induced Mitochondrial Injury and Cell Apoptosis

Building upon the prior findings that established a link between reduced mitophagy and cellular inflammation, apoptosis, and necrosis, our study further investigates the consequences of NR1D1 deficiency, a condition associated with impaired mitophagy. Importantly, the pharmacological activation of NR1D1 using SR9009 was found to upregulate NR1D1 expression levels, as validated by Western blot analysis (Figure S4).

**Figure 8:**
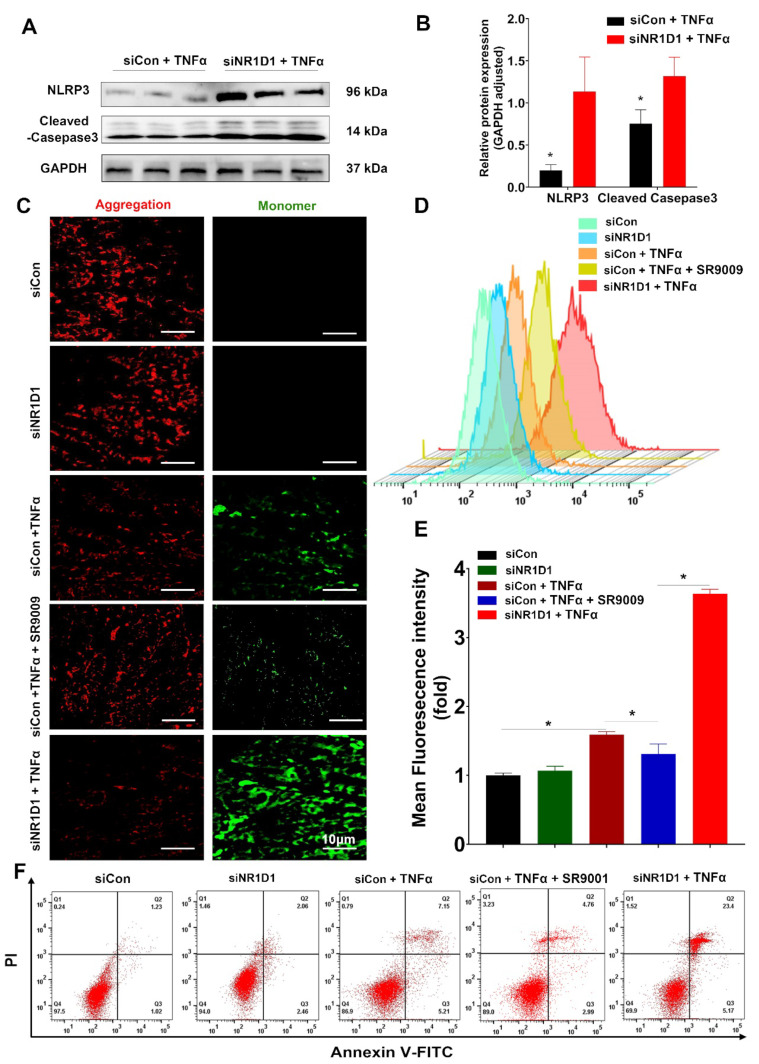
SR9009 activation of NR1D1 mitigates TNFα-induced cellular pathologies. (**A**,**B**) Immunoblotting along with semi-quantitative densitometric evaluation for assessing the protein expression of NLRP3 and cleaved caspase-3. (**C**) Representative confocal images illustrating MMP changes, assessed via the JC-1 aggregate:monomer ratio, demonstrate resilience to TNFα-induced MMP loss upon SR9009 treatment, scale bar = 10 μm. (**D**,**E**) Flow cytometric quantification of ROS via DCFH-DA in CCD841 CoN cells, either siCon- or siNR1D1-pre-treated and TNFα-stimulated, shows ROS attenuation upon SR9009 treatment. (**F**) Apoptosis in CCD841 CoN cells, assessed by flow cytometry following siCon or siNR1D1 and TNFα treatment, is mitigated by SR9009 intervention. Data are presented as means ± standard deviations (*n* = 3–6). * *p* < 0.05, as determined by Student’s *t*-test.

In NR1D1 KD cells, we observed an exacerbated expression of NLRP3 and cleaved caspase-3 upon TNFα stimulation (Figure 8A,B). In contrast, cells treated with SR9009 exhibited a less pronounced decline in mitochondrial membrane potential (MMP), as evidenced by the reduced alteration in JC-1 aggregates and monomers following TNFα stimulation (Figure 8C). Similarly, SR9009 treatment mitigated the increase in cellular ROS levels observed upon TNFα stimulation in NR1D1-deficient cells (Figure 8D,E). Complementing these findings, a flow cytometry analysis indicated that SR9009 treatment resulted in a reduced rate of cell apoptosis under TNFα stimulation compared to NR1D1 KD cells (Figure 8F).

Collectively, these results underscore the crucial role of NR1D1 in modulating mitophagy and indicate that its pharmacological activation via SR9009 can mitigate TNFα-induced cellular damage. This study, therefore, highlights the potential of NR1D1 as a therapeutic target for conditions associated with impaired mitophagy and cellular stress.

### 2.6. Amelioration of DSS-Induced Colitis in Mice by the NR1D1 Agonist SR9009

**Figure 9:**
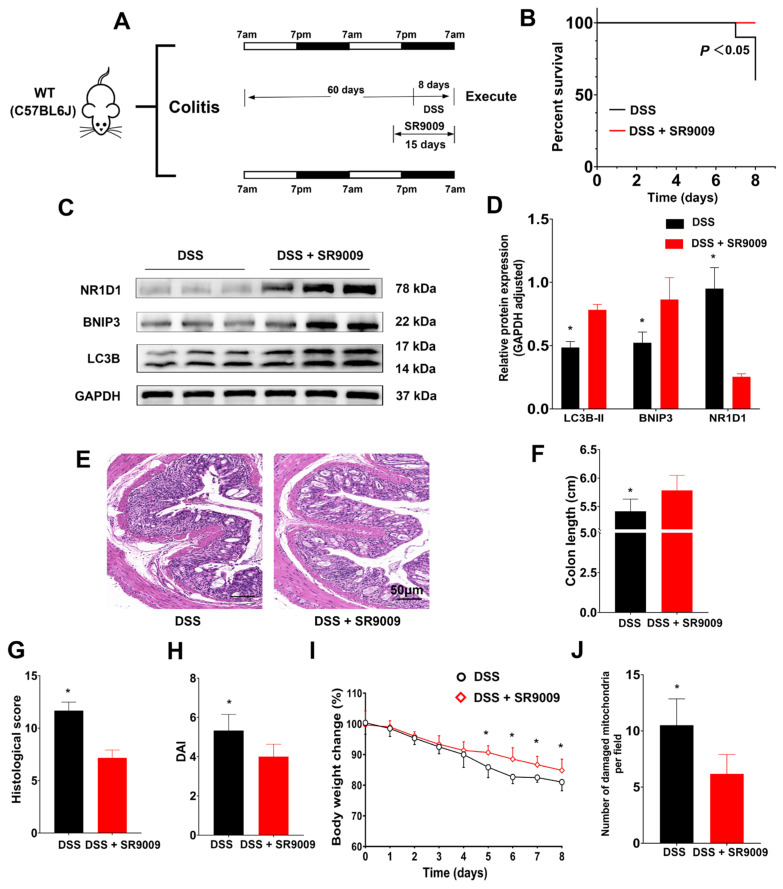
SR9009 alleviates symptoms. (**A**) Schematic representation of the colitis mouse model. (**B**) Survival rate of mice subjected to DSS-induced colitis and treatment with SR9009. (**C**,**D**) Protein expression levels of NR1D1, BNIP3, and LC3B-II in intestinal epithelial cells (IECs). (**E**) Histological evaluation of colon tissue, scale bar = 50 μm. (**F**–**I**) Comparisons of colon length, histological scores, DAI scores, and body weight loss among the experimental groups. (**J**) Quantification of damaged mitochondria in IECs. Data are presented as means ± standard deviations (*n* = 3–6). * *p* < 0.05 (Student’s *t*-test).

To authenticate the influence of NR1D1 in the context of UC, we employed animal experiments utilizing the NR1D1 agonist SR9009. Mice received daily intraperitoneal injections of SR9009 (50 mg/kg/day) from day 45 to 60 and were concurrently exposed to DSS from day 52 to 60 (Figure 9A). In response to SR9009 administration, the survival rates notably improved among DSS-treated mice (Figure 9B). Further, the treatment induced an upsurge in NR1D1 protein expression and concurrently elevated BNIP3 and LC3B-II protein levels within IECs (Figure 9C,D). Significantly, treatment with SR9009 enhanced colon length, mitigated histological scores, decreased DAI scores, and curtailed body weight loss in mice with colitis, while notably also reducing the count of damaged mitochondria (Figure 9E–J). Collectively, these findings substantiate the role of SR9009 in augmenting NR1D1 expression and subsequently mitigating the severity of DSS-induced UC in mice.

## 3. Discussion

**Figure 10:**
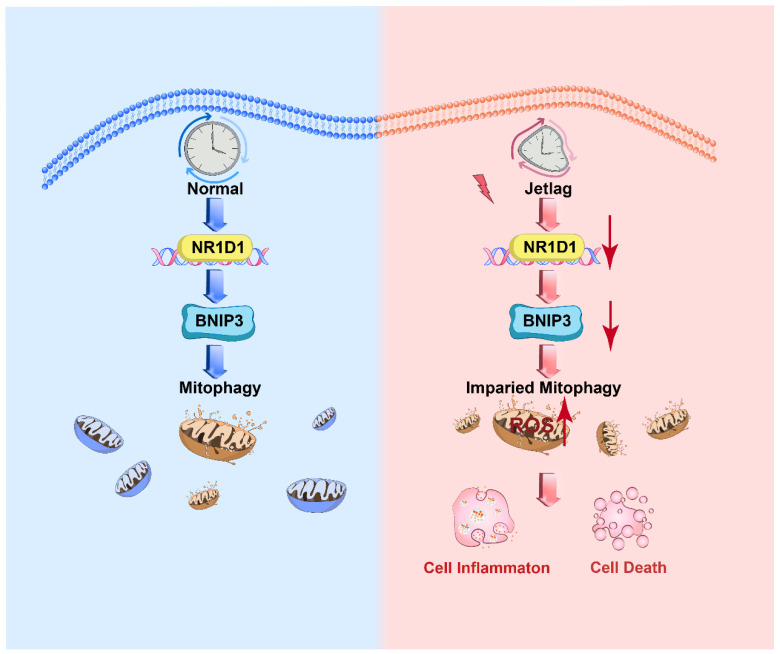
NR1D1, BNIP3, and mitophagy interplay in jet lag and colitis. Jet lag and colitis are hypothesized to cause a decrease in NR1D1 expression in IECs, leading to a concomitant reduction in BNIP3 expression and subsequent impairment of mitophagy. This series of events results in an upregulation of NLRP3 and cleaved caspase-3 expression, driving mitochondrial dysfunction as characterized by a decrease in MMP and an increase in ROS levels. Collectively, these changes promote cellular inflammation and apoptosis, illustrating the complex interplay between circadian disruption, gene expression, and cellular responses in the pathogenesis of jet lag and colitis.

In this investigation, we examined the influence of NR1D1 on mitophagy in IECs amidst the context of UC. We engineered mouse models simulating both jet lag and colitis, enabling the observation of alterations in the expression dynamics of clock genes within IECs, specifically the notable downregulation of NR1D1. In vivo evidence revealed that the absence of NR1D1 aggravated the severity of DSS-induced colitis, establishing NR1D1 as a pivotal nexus between circadian rhythms and UC manifestation. UC is often characterized by persistent inflammation and IEC damage [36], with mitochondria playing a crucial role in regulating IEC homeostasis [10]. Through transmission electron microscopy, we observed an elevated quantity of damaged mitochondria within IECs. NR1D1 functions as a transcriptional activator, binding to BNIP3’s promoter region and driving its transcription. Consequently, a reduction in NR1D1 expression triggers a decrease in BNIP3 expression, thereby impeding mitophagy, exacerbating inflammation, and accelerating cell apoptosis within IECs (Figure 10).

Due to the escalation in international travel, shift work, social engagements, and life stressors, circadian rhythm disturbances are increasingly prevalent. These disturbances may perturb the expression of circadian clock genes [37]. Notably, clock genes capture our interest as they orchestrate immune function, energy metabolism, and mitophagy [25,38,39], thus exerting diverse impacts on mammalian physiology. However, the mechanisms by which the circadian clock affects UC remain unclear. Alterations in circadian clock gene expression have been shown to trigger the development of IBD [40], and the loss of clock genes exacerbates DSS-induced colitis in mice [41], which corresponds with our findings (Figure 4B–F). Notably, previous research on the circadian clock and UC often employed the entire intestine of mice rather than IECs [42,43,44]. However, UC is characterized by superficial inflammation infiltration and damage to IECs. Therefore, by focusing our experiments on IECs and minimizing the influence from other intestinal cells, we can gain a more precise understanding of the role clock genes play within IECs. 

After establishing the mouse models, we isolated the IECs and discovered that jet lag and colitis impacted the expression pattern of clock genes, resulting in reduced NR1D1 expression. Additionally, the RNA sequencing of IECs revealed that DEGs in the mouse model of jet lag were enriched in several infection- and immune-related pathways (Figure 3B–D). Consequently, we hypothesized that the clock gene NR1D1 served as a crucial regulator of UC, and we explored the mechanisms that underlie this relationship. The selection of NR1D1 as our primary target gene for this study is further justified by the observed reduction in its expression levels. This reduction aligns well with its diminished expression in other conditions affecting immune homeostasis, such as jet lag. Moreover, given NR1D1’s pivotal role in regulating the immune environment within IECs, it emerges as a promising therapeutic target for UC. Future studies could substantively extend our findings by exploring the direct impact of NR1D1 expression modulation on UC pathophysiology.

As a critical aspect of mitochondrial quality control, mitophagy sustains cellular and mitochondrial homeostasis by removing damaged mitochondria. Notably, mitochondrial dysfunction is common in UC patients. Previous studies have indicated that optimal mitophagy can uphold the balance in IECs and alleviate colitis symptoms. Crucially, if mitophagy is impaired, it can amplify stress-induced cellular damage, thereby stimulating cellular inflammation and apoptosis [26,45,46], observations that align with our findings. 

Upon interpreting initial ChIP-seq data, we proceeded with ChIP and dual-luciferase assays. Together, these analyses disclosed that NR1D1 heightens BNIP3 transcription by directly interacting with its promoter. This evidence serves to establish a novel regulatory link between NR1D1, a clock gene implicated in UC, and BNIP3, a pivotal mediator of mitophagy. Our ChIP-seq data initially hinted at this transcriptional control, which was then robustly validated through further ChIP and dual-luciferase experiments. This adds substantial evidence suggesting that NR1D1 could modulate the pathological landscape in UC by directly controlling BNIP3-mediated mitophagy. Given BNIP3’s role in PRKN-independent mitophagy, this discovery adds a new layer to our understanding of how clock genes like NR1D1 can intricately affect mitochondrial dynamics and, consequently, the pathophysiology of UC.

Our in vitro experiments demonstrated that NR1D1 KD augments the expression of NLRP3 and cleaved caspase-3 after TNFα stimulation. Given the continual damage to mitochondria inflicted by NLRP3 and the role of cleaved caspase-3 as an apoptotic marker [45], compromised mitophagy can thus exacerbate cellular damage. Moreover, TNFα exposure culminated in a decline in mitochondrial membrane potential and a surge in ROS within the IECs subjected to NR1D1 KD. The flow cytometry assay further corroborated that NR1D1 KD amplifies TNFα-induced apoptosis. Collectively, these results provide insights into the increased prevalence of damaged mitochondria in IECs and the disruption in immune homeostasis induced by NR1D1 depletion.

In our investigations involving IECs, we discerned that NR1D1 modulates mitophagy via the positive regulation of BNIP3, a pivotal gene harmonizing the process of mitophagy. Our results have highlighted a decrease in BNIP3 expression following the KD of NR1D1. Given that NR1D1 is typically a repressor [46], the precise mechanism through which its knockdown influences BNIP3 remains a puzzle, leading to two plausible hypotheses. One postulates that NR1D1 could negatively regulate an inhibitor of BNIP3, whereas the alternative conjecture posits that NR1D1 might enhance BNIP3 gene regulation potentially through interaction with a specific modulator. Notably, there exist prior studies supporting the potential of NR1D1 to positively modulate gene transcription via either of these mechanisms [47,48,49].

IECs constitute the main physical partition separating the intestinal microbiota and content from the host tissue and play a pivotal role in regulating intestinal immune homeostasis [5]. Prior research indicates that IEC damage amplifies UC severity, a finding that is corroborated by our study [50,51]. Consequently, minimizing the inflammatory infiltration of IECs and maintaining their homeostasis is crucial for IBD prevention and treatment. Circadian rhythm perturbations result in diminished NR1D1 expression in IECs, potentially disrupting immune homeostasis within these cells. Moreover, an investigation into the NCBI Gene Expression Omnibus Database reveals diminished NR1D1 expression in a significant subset of UC patients, even outside circadian rhythm disruptions. This suggests that substantial portions of UC patients could suffer impaired mitophagy in IECs due to reduced NR1D1 expression. Future research holds the potential to reinforce these observations. Nonetheless, our study posits NR1D1 as a promising therapeutic target for UC. 

While the pathogenesis of UC continues to be elusive, treatment regimens including 5-aminosalicylic acid, hormones, immunosuppressive drugs, and biological agents are typically employed, based on disease severity. Regrettably, a substantial subset of patients exhibit unresponsiveness to these medications, rendering the therapeutic landscape for UC noticeably barren. As such, the quest for innovative treatments for UC is crucial. Our investigation provides evidence that the NR1D1 agonist SR9009 promotes survival during colitis, restricts excessive mitophagy, and ameliorates UC in murine models. Beyond its role in modulating mitophagy, SR9009 could potentially dampen inflammation via diverse pathways. Prior studies by Pourcet et al. have shown that SR9009 mitigated acute peritoneal inflammation and severe hepatitis in mouse models [52]. Moreover, SR9009 attenuated neuronal damage in the hippocampus post-status epilepticus [53], and SR9009 induced macrophage polarization from M1 to M2 (anti-inflammatory) [54], thereby reducing atherogenesis [55]. This array of findings substantiates the anti-inflammatory potential of NR1D1 and, notably, our work elucidates a novel mechanism through which it may exert this effect.

In conclusion, our study highlights the integral role of the circadian rhythm gene NR1D1 in orchestrating BNIP3-mediated mitophagy and thus maintaining the homeostasis of IECs. We uncovered that NR1D1 deficiency curtails mitophagy, thereby escalating inflammation and apoptosis in IECs during UC. Importantly, we recognized the NR1D1 activator, SR9009, as a promising candidate for UC therapy. This investigation not only sheds light on novel aspects of UC pathogenesis but also underscores the potential of the NR1D1–BNIP3–mitophagy axis as a viable target for IBD intervention. The need for a deeper exploration of the intricate relationship between circadian biology, mitophagy, and inflammatory disorders is underscored by our findings, with the goal of enhancing therapeutic approaches for conditions like IBD.

## 4. Materials and Methods

Antibodies, reagents, and plasmids: for immunoblotting, antibodies against NR1D1 [#13418S], BMAL1 [#14020S], CLOCK [#5157S], cleaved caspase-3 [#9664S], and GAPDH [#5174S] (Cell Signaling Technology; Danvers, MA, USA); CRY1 [#ab171860], NLRP3 [#ab263899] (Abcam; Cambridge, UK); CRY2 [#13997-1-AP], PER1 [#13463-1-AP] (Proteintech; Wuhan, China); PER2 [#A5107] (ABclonal; Wuhan, China); LC3B [#L7543] (Sigma-Aldrich; St. Louis, MI, USA); BNIP3 [#sc-56167] (Santa Cruz; Santa Cruz, CA, USA).

Flow cytometry was performed using antibodies against annexin V-fluorescein isothiocyanate [#C1062L] (Beyotime; Shanghai, China). The reactive oxygen test kit [#S0033S] was purchased from Yeasen (Shanghai, China).

Immunohistochemistry was performed using the anti-NR1D1 antibody [#13418S] (Cell Signaling Technology; Danvers, MA, USA). Immunofluorescence was performed using antibodies against LC3B [#L7543] (Sigma-Aldrich; St. Louis, MI, USA), BNIP3 [#sc-56167] (Santa Cruz; Santa Cruz, CA, USA), Lamp2 [#ab125068], and VDAC [#ab14734] (Abcam; Cambridge, UK). Apoptosis in colonic tissues was detected via terminal deoxynucleotidyl transferase-mediated dUTP nick-end labeling (TUNEL) (Beyotime; Shanghai, China).

Chromatin immunoprecipitation (ChIP) was performed using anti-NR1D1 [#13418S] and anti-rabbit IgG [#14708S] (Cell Signaling Technology; Danvers, MA, USA).

MitoTracker Red [#M7521], MitoTracker Green [#M7514], and LysoTracker Red [#L12492] were purchased from Thermo Fisher Scientific (Waltham, MA, USA).The Enhanced Mitochondrial Membrane Potential Assay Kit with JC-1 [#M354215] was obtained from Thermo Fisher Scientific (Waltham, MA, USA).

Dextran sulfate sodium (DSS) [#9011-18-1] was purchased from MP Biomedicals (Solon, OH, USA). SR9009 [#554726] was obtained from Calbiochem (San Diego, CA, USA). TNF-α [#300-01A] was purchased from Peprotech (Rocky Hill, CT, USA). The ChIP kit [#17295] was purchased from Sigma-Aldrich (St. Louis, MI, USA). The Dual-Luciferase Reporter Assay System [#E1910] was obtained from Promega (Madison, WI, USA). CCD 841 CoN cells were purchased from ATCC (Gaithersburg, MD, USA). The Lipofectamine™ RNAiMAX Transfection Reagent [#13778150] and the Lipofectamine LTX with PLUS Reagent [#15338100] were obtained from Thermo Fisher Scientific (Waltham, MA, USA). All reagents for q-PCR were purchased from Qiagen (Hilden, Germany), QIAzol [#79306], QuantiTect Primer Assay [#249900], and QuantiFast SYBR Green [#204056]. The ELISA kits for TNF-α [#PT513], IL-1β [#PI301], IL-17 [#PI545], and IL-6 [#PI326] were purchased from Beyotime (Shanghai, China).

A small interfering RNA (siRNA) specifically targeting NR1D1 and a pcDNA control vector, as well as a recombinant plasmid for the overexpression of BNIP3, were synthesized and supplied by Obio Technology (Shanghai, China). The pRL Renilla luciferase control reporter vectors and PGL4.11 vectors were purchased from Promega (Madison, WI, USA). The sequencing of all plasmids confirmed their sequences.

Patient tissue collection: Endoscopic biopsy specimens were collected from 6 patients with UC, along with 6 patients undergoing colonoscopies for non-inflammatory and non-IBD conditions. Following collection, a portion of each specimen was preserved in paraformaldehyde for immunofluorescence staining after dissection. The remaining samples were analyzed using qPCR, with snap-frozen specimens stored at −80 °C. Informed consent was obtained from all patients, and the study received approval from the Independent Ethics Committee of Wuhan Union Hospital.

Animals: Wild-type (WT) C57BL/6 mice were acquired from Beijing HFK Bioscience (Beijing, China). Intestinal epithelial cell (IEC)-specific Nr1d1 knockout (KO) mice, referred to as Nr1d1^−/−^ in subsequent text, were generated from Nr1d1fl/fl-Villin-Cre mice on a C57BL/6 background using the CRISPR/Cas9 system (Cyagen Biosciences Inc.; Suzhou, China). The animals were provided with ad libitum access to standard chow and water. The study received approval from the Research Ethics Committee of Tongji Medical College at the Huazhong University of Science and Technology.

Male mice aged 7–10 weeks were utilized for the experiments. In the jet lag mouse model, animals were housed in standard cages under a 12/12 h light–dark cycle (lights on/off at 07:00/19:00). To simulate conditions akin to shift work, particularly in healthcare professionals who experience frequent circadian disruption, our mice underwent a more severe 12 h time shift every 3 days. This schedule was maintained over a 60-day period. This design contrasts with previous studies that generally employ an 8 h light cycle advance every 23 days [56]. Acute colitis was induced by administering 3% DSS in tap water (*w*/*v*) for 8 days, as per established protocol [57]. To assess the effect of SR9009 on UC, mice were intraperitoneally injected with SR9009 (50 mg/kg) once daily from day 45 to 60.

Mice colon tissue collection: Mice were euthanized at various zeitgeber times (ZT), specifically at ZT2, ZT6, ZT10, ZT14, ZT18, and ZT22, with 6 mice from each group euthanized at each time point. The colonic tissue was carefully removed, with a section cut 0.5 cm from the distal colon. This tissue was then fixed in paraformaldehyde and embedded in paraffin in preparation for subsequent procedures such as HE staining, immunohistochemistry, transmission electron microscopy, and immunofluorescence. The remaining colonic tissue was employed for the isolation of IECs.

Histology, immunohistochemistry, and immunofluorescence: Colon tissues were paraffin-embedded, sectioned to a thickness of 3 µm, and stained with hematoxylin-eosin (HE). Two pathologists blinded to group allocation scored HE-stained sections as described previously [58]. 

In terms of immunohistochemistry, the sections were first deparaffinized and subjected to antigen retrieval. Following this, they were incubated with hydrogen peroxide in the dark for 30 min to quench endogenous peroxidase activity. Tissue sections were then incubated overnight at 4 °C with the primary antibody against NR1D1. On the subsequent day, after washing with phosphate-buffered saline (PBS), sections were exposed to horseradish peroxidase-conjugated secondary antibodies for 1 h at room temperature. Peroxidase activity was visualized using 3,3′-diaminobenzidine. The tissue sections were subsequently dehydrated using a graded ethanol series before mounting. The expression of NR1D1 was evaluated under a light microscope and quantified using the Fiji software version 1.5.1 (Bethesda, MD, USA).

For immunofluorescence analysis, colon sections were treated with primary antibodies (all diluted 1:200) and incubated overnight at 4 °C, followed by an additional 1 h incubation at room temperature the following day. After three washes with PBS, the sections were stained with DAPI to mark the nuclei after incubation with fluorescent secondary antibodies for 1 h and subsequent PBS washes. The terminal deoxynucleotidyl transferase-mediated dUTP nick-end labeling (TUNEL) assay was carried out as per the manufacturer’s guidelines. 

For cellular investigations, CCD841 CoN cells were cultivated in confocal dishes, with both mitochondria and lysosomes duly labeled. The cells underwent treatment with either MitoTracker Red (500 nM) or MitoTracker Green (50 nM) for mitochondrial staining, LysoTracker Red (50 nM) for lysosomal staining, and Hoechst (5 μg/mL) for live cell nuclear staining, all in accordance with the manufacturer’s guidelines. Following treatment with MitoTracker Red, cells were fixed with formaldehyde. Subsequent antibody incubation was executed following the same protocol as employed for colon sections. Upon fixation, nuclei were restained using DAPI, facilitating the imaging of cells on a Zeiss confocal microscope (Jena, Germany). 

Transmission electron microscopy: Colon tissues were thoroughly rinsed with cold PBS and transversely sectioned into 5 mm slices. These sections were fixed overnight in 4% paraformaldehyde, followed by a 2 h incubation with 1% osmium tetroxide at room temperature. After three rounds of washing in PBS, the sections were subjected to dehydration via a graded ethanol series and subsequently embedded in epoxy resin. Serial ultra-thin sections, approximately 80 nm in thickness, were then prepared. The mitochondria within IECs were examined using a transmission electron microscope (Model HT7800; Hitachi, Tokyo, Japan).

Isolation of IECs: IECs were extracted following a previously detailed protocol [59]. Mouse colon samples were thoroughly washed thrice with Dulbecco’s PBS (DPBS) at 4 °C and sectioned into 0.5 cm slices. These tissue sections were incubated in a buffered solution (comprising 14 mL of DPBS, 0.9 mL of 0.5 M EDTA, and 22.5 µL of 1 M DTT) for 75 min and centrifuged. The supernatant was then carefully removed, and the pellet was resuspended in 5 mL of the buffered solution via vortexing. This centrifugation and resuspension process was repeated three times to maximize IEC recovery. The resulting cell suspension was filtered through a 40 μm cell strainer to procure a purified IEC population. The isolated IECs were then employed in downstream applications such as qPCR, Western blotting, RNA-seq, and ELISA for the examination of gene expression, protein abundance, and transcriptomic profiles, respectively.

Cytokine Quantification by ELISA: Isolated IECs were subjected to lysis and protein concentration was ascertained via the Bradford assay. Aliquots were stored at −80 °C until assayed. ELISA kits were deployed for quantifying TNF-α, IL-1β, IL-10, and IL-17 according to the manufacturer’s guidelines. Briefly, 100 μL of sample or standard was incubated in cytokine-specific antibody-coated wells (2–3 h, room temperature, gentle shaking). After three washes with buffer, 100 μL of detection antibody was added (1 h, room temperature). Subsequent to washing, 100 μL of substrate solution was added, and plates were incubated in the dark (15–30 min). Reactions were quenched by adding 50 μL of stop solution. Absorbance at 450 nm was measured using a microplate reader.

Cell Culture and Treatment: CCD 841 CoN cells were cultured under standard conditions in a humidified incubator at 37 °C and 5% CO_2_. The growth medium was supplemented with 10% fetal bovine serum and 1% penicillin-streptomycin. For induction of apoptosis and mitochondrial damage, cells were exposed to TNFα at a concentration of 10 ng/mL for a duration of 24 h. Pharmacological intervention was performed using SR9009 at a concentration of 20 μM, with the treatment period lasting 48 h.

siRNA-Mediated Knockdown and Transfection: For the targeted silencing of NR1D1, CCD841 CoN cells were transfected with pre-synthesized siRNA specific to NR1D1 or a pcDNA control vector. Transfections were conducted using Lipofectamine RNAiMAX Transfection Reagent, following the manufacturer’s protocol. Opti-MEM medium was utilized during the transfection process. Post-transfection, cells were incubated for 48 h to ensure effective gene silencing, after which they were harvested for downstream analyses, including qPCR and Western blot assays.

Overexpression of BNIP3: For the forced overexpression of BNIP3, CCD841 CoN cells were transfected with a recombinant plasmid encoding the full-length cDNA of BNIP3. Transfection was performed using Lipofectamine LTX with PLUS Reagent, as per the manufacturer’s guidelines. Opti-MEM medium served as the transfection medium. Following a 48 h post-transfection incubation period, cells were harvested for subsequent assays.

Western blotting: Cells were lysed in RIPA buffer containing 1× protease and phosphatase inhibitor cocktail. The lysates were sonicated and centrifuged at 12,000× *g* for 15 min at 4 °C. The supernatant was collected, and protein concentration in the supernatant was measured using the bicinchoninic acid assay. Protein samples were separated by sodium dodecyl sulfate-polyacrylamide gel electrophoresis and transferred to polyvinylidence difluoride membranes. Membranes were blocked with skim milk for 1–3 h, placed on a shaker at 4 °C, and incubated with primary antibody overnight. The next day, the secondary antibody was incubated for 1 hr after using the membranes washed with TBST. Blots were visualized using a chemiluminescence detection system and quantitated using Fiji version 1.5.1 (Bethesda, Rockville, MD, USA). GAPDH was used as a loading control.

Quantitative real-time polymerase chain reaction (qPCR): RNA was extracted using QIAzol. The concentration of RNA was determined using a spectrophotometer, and subsequently, the RNA was reverse-transcribed into cDNA. Gene expression levels were quantified with qPCR on a LightCycler 480 fluorescent quantitative PCR system (Roche Diagnostics, Indianapolis, IN, USA). The relative expression of each gene was normalized against GAPDH using the 2-ΔΔCT method. The primer design was performed using Primer Premier 6.0 software (PREMIER Biosoft International, Palo Alto, CA, USA). The specific primer sequences are presented in Table 1.

RNA sequencing: Total RNA was extracted from IECs, and its concentration was assessed using a NanoDrop spectrophotometer. RNA integrity was verified with an Agilent 2100 Bioanalyzer (Agilent Technologies, Santa Clara, CA, USA). RNA-seq libraries were prepared using 2 μg of RNA from each sample and the NEBNext mRNA Sample Prep Kit. The libraries were sequenced on the Illumina NovaSeq 6000 platform. Transcriptome data were provided by Frasergen (Wuhan, China). Differentially expressed genes (DEGs) were identified by comparing gene expression between groups using DESeq2 version 1.10.1. DEGs were defined as having |log2(fold change)| > 2 and a corrected *p*-value less than 0.05.

Flow cytometry: Both WT CCD 841 CoN cells and NR1D1 KD CCD 841 CoN cells underwent treatment with NR1D1 siRNA and TNFα. Post-treatment, we employed annexin V-fluorescein isothiocyanate staining to discern phosphatidylserine externalization, indicative of early apoptotic events. Moreover, intracellular ROS levels, serving as indicators of cellular damage, were measured using the fluorescent probe dichlorofluorescein diacetate. Data collection was performed via flow cytometry, and the resulting data were subsequently analyzed using FlowJo software (version 10.8.1).

Mitochondrial membrane potential detection (MMP): MMP was assessed using JC-1 staining. Cells were centrifuged, resuspended in JC-1 working solution, and incubated for 20 min. After incubation, the cells were washed twice with JC-1 buffer. Confocal microscopy was employed to detect fluorescence intensity, with green and red fluorescence (corresponding to JC-1 monomers and JC-1 aggregates, respectively) observed using 488 nm and 594 nm filters.

Luciferase assays: Luciferase assays were carried out using the luciferase reporter vector pGL4.11-Basic, engineered to contain either the wild-type (WT) or a 7 base-pair mutation in the promoter region of BNIP3, resulting in vectors termed pGL4.11/WT and pGL4.11/Mut, respectively. Cells were transfected with these reporter plasmids, along with pRL Renilla luciferase control reporter vectors and pcDNA containing the NR1D1 plasmid or an empty vector serving as a control. Subsequently, the dual-luciferase activity was quantified using a Promega luminometer (Madison, USA).

Chromatin immunoprecipitation (ChIP)-qPCR: CCD 841 CoN cells were fixed with formaldehyde, and cross-linked protein-DNA complexes were prepared using a ChIP kit. Complexes were incubated with a primary antibody against NR1D1 or rabbit IgG for 18 h at 4 °C with gentle agitation. Magnetic beads were added with gentle agitation for 2 h. DNA was eluted from the beads. Primers flanking the NR1D1 binding site on the human BNIP3 promoter were used in qPCR assays, and PCR results were visualized using agarose gel electrophoresis. The primers for human BNIP3 were 5′-TCCCAAAGTGCTGAGATGAAAGAC-3′ (forward) and 5′-CTCTTGCCTGTTGAATCGTCTCTC-3′ (reverse).

Statistical analysis: Data are presented as means ± standard deviations. Statistical significance was analyzed using an unpaired two-tailed Student’s *t*-test, a non-parametric test (Mann–Whitney U test), and a one-way analysis of variance. A *p*-value of less than 0.05 was considered statistically significant.

## Figures and Tables

**Table 1:** Primer sequences used for qPCR.

Gene	Sequence
Mouse *Nr1d1*	F:ACTTCCCACCATCACCTACTG
	R:GGGGAGCTATCATCACTGAGA
Mouse *Clock*	F:ATGGTGTTTACCGTAAGCTGTAG
	R:CTCGCGTTACCAGGAAGCAT
Mouse *Bmal1*	F:ACAGTCAGATTGAAAAGAGGCG
	R:GCCATCCTTAGCACGGTGAG
Mouse *Cry1*	F:CACTGGTTCCGAAAGGGACTC
	R:CTGAAGCAAAAATCGCCACCT
Mouse *Cry2*	F:CACTGGTTCCGCAAAGGACTA
	R:CCACGGGTCGAGGATGTAGA
Mouse *Per1*	F:GAATTGGAGCATATCACATCCGA
	R:CCCGAAACACATCCCGTTTG
Mouse *Per2*	F:CTCCAGCGGAAACGAGAACTG
	R:TTGGCAGACTGCTCACTACTG
Mouse *Gapdh*	F:AGGTCGGTGTGAACGGATTTG
	R:GGGGTCGTTGATGGCAACA
Human *NR1D1*	F:TGGACTCCAACAACAACACAG
	R:GATGGTGGGAAGTAGGTGGG
Human *GAPDH*	F:GGAGCGAGATCCCTCCAAAAT
	R:GGCTGTTGTCATACTTCTCATGG

## Data Availability

The data supporting this study’s findings were openly available in the NCBI SRA database (https://dataview.ncbi.nlm.nih.gov/object/PRJNA818060) (accessed on 1 August 2023).

## Supplementary Materials

The following supporting information can be downloaded at: https://www.mdpi.com/article/10.3390/ijms241814222/s1.
