# Supplementary material for: Uncovering the Novel Role of NR1D1 in Regulating BNIP3-Mediated Mitophagy in Ulcerative Colitis
Source: Int J Mol Sci. 2023 Sep 18;24(18):14222. doi: 10.3390/ijms241814222 (PMC10531686; doi:10.3390/ijms241814222)
Supplement: Supplementary file 1 [file ijms-24-14222-s001.zip › ijms-2575368 supplementary/ijms-2575368 Supplementary Figure S1-S4.pdf]

Supplementary Figure S1:

A

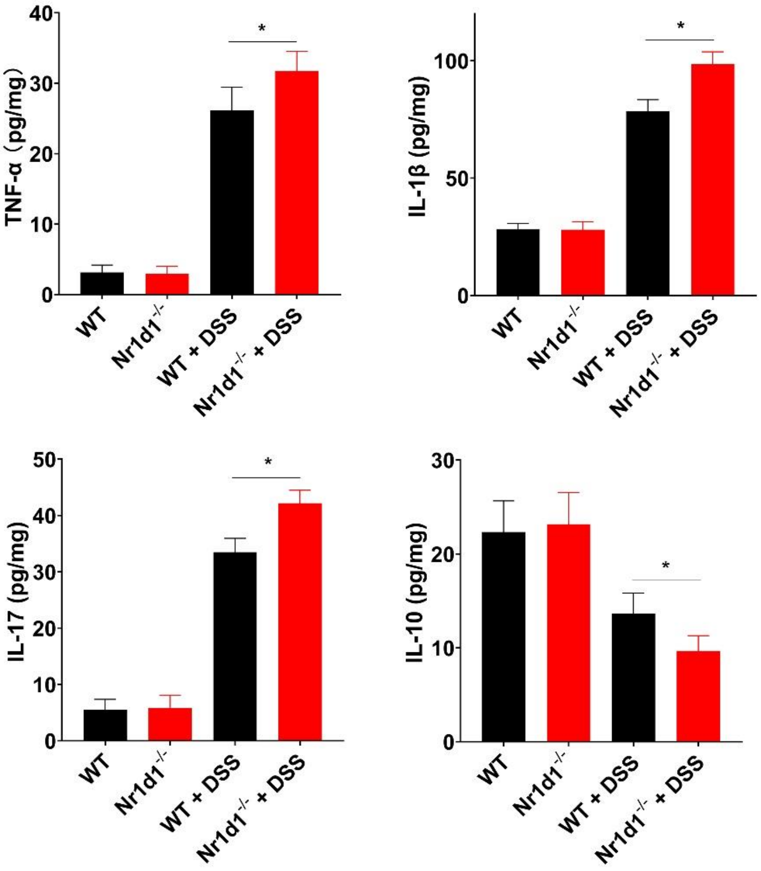

Elevated Inflammatory Cytokine Levels in NR1D1-Deficient Mouse IECs Under Colitis Conditions

(A) ELISA quantification of cytokines TNFα (a), IL-6 (b), IL-1β (c), and IL-10 (d) in intestinal epithelial cells (IECs) isolated from wild-type (WT) and NR1D1-deficient mice, under both standard and colitis-inducing conditions. Four experimental groups are represented: WT, NR1D1 knockout (KO), WT with induced colitis, and NR1D1 KO with induced colitis. Cytokine concentrations are expressed in pg/mL. Data points are mean ± SEM, n=6 per group.Asterisks indicate statistically significant differences (*$P < 0.05$).

Supplementary Figure S2:

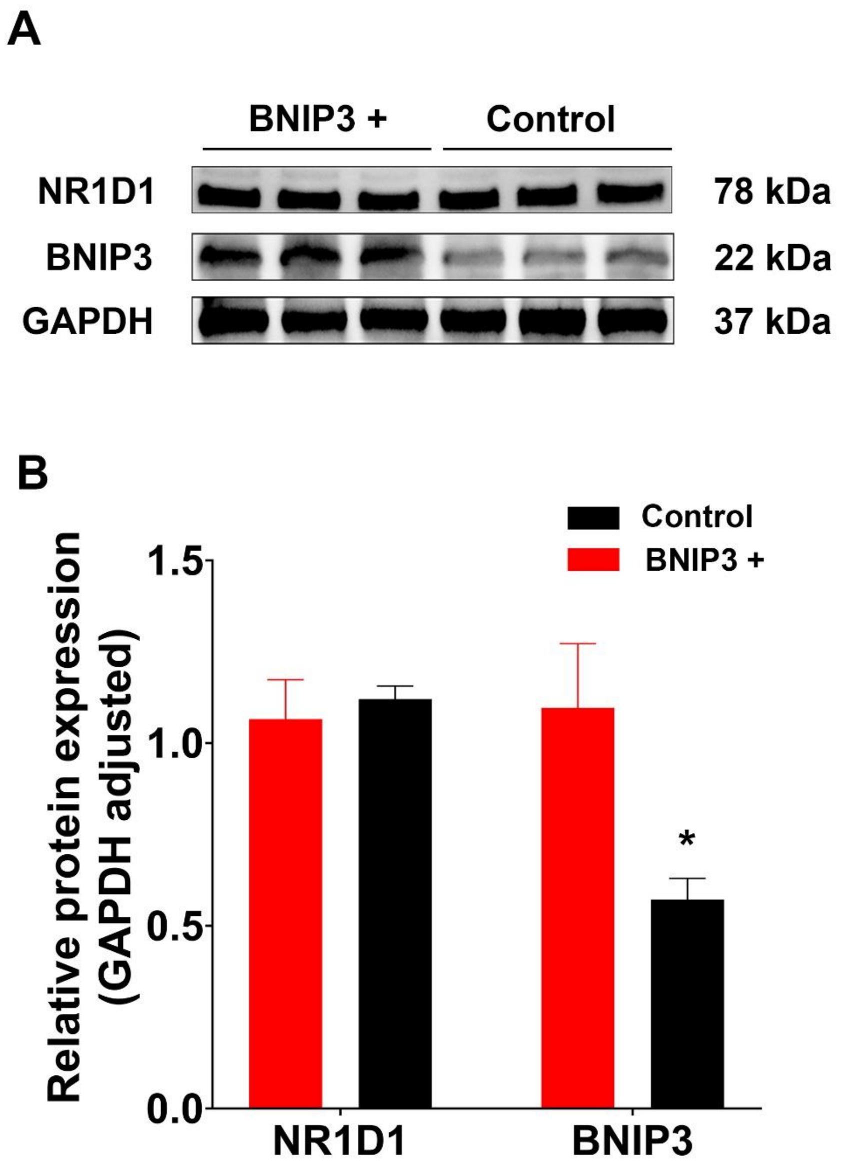

Overexpression of BNIP3 Does Not Affect NR1D1 Protein Levels

(A) Representative Western blot images of BNIP3 and NR1D1 protein levels, using GAPDH as an internal control. Two experimental conditions are shown: Control and BNIP3-overexpressed (BNIP3+). (B) Quantification of relative protein expression for BNIP3 and NR1D1, normalized to GAPDH levels. Expression levels are further normalized to the Control group. Data points represent mean ± SEM, n=3 per group. Asterisks indicate statistically significant differences in BNIP3 levels between Control and BNIP3+ groups (*$P < 0.05$).

Supplementary Figure S3:

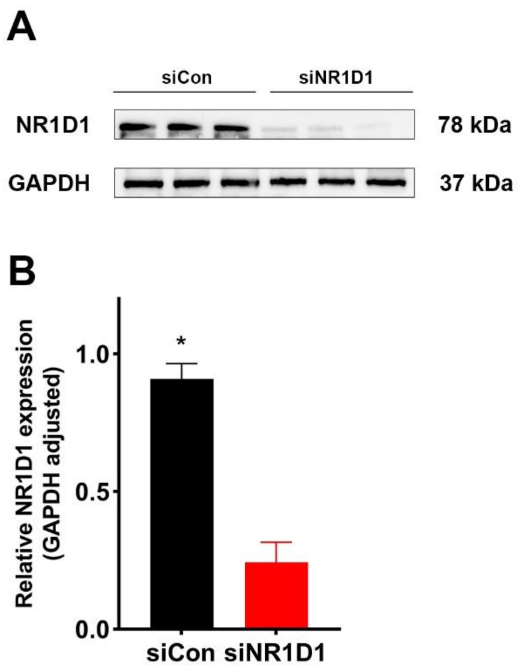

Validation of NR1D1 knockdown in CCD841 CoN cells

(A) Western blot analysis of CCD841 CoN cells transfected with either si-NR1D1 or si-Control, showcasing protein levels of NR1D1 and the housekeeping gene GAPDH.

(B) Quantitative analysis of the relative protein expression levels from the western blot results in (A). Data are represented as mean ± SEM from three independent experiments (n=3). The asterisk (*) indicates a significant difference with $p < 0.05$.

Supplementary Figure S4:

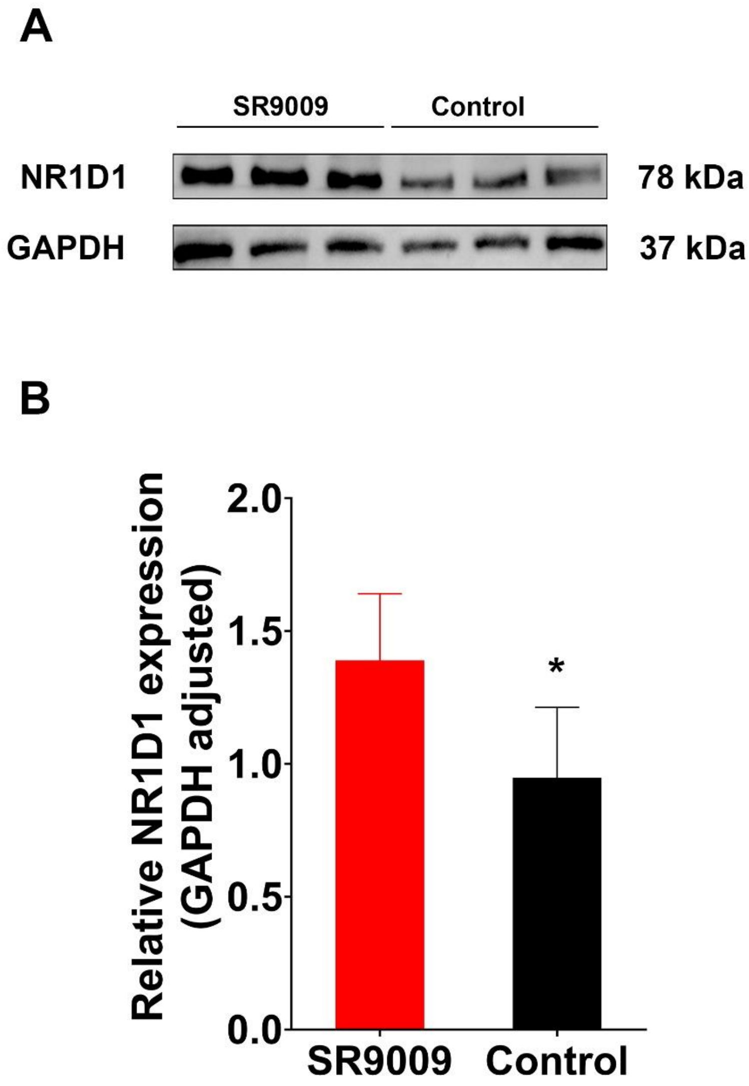

Effect of SR9009 Treatment on NR1D1 Expression

(A) Representative Western blot images displaying the protein levels of NR1D1, with GAPDH serving as an internal control. Two experimental conditions are assessed: Control and SR9009-treated. (B) Quantification of relative protein expression levels of NR1D1, normalized to GAPDH levels. Expression levels are further normalized to the Control group. Data points represent mean ± SEM, n=3 per group. Asterisks indicate statistically significant differences between the Control and SR9009-treated groups (*$P < 0.05$).
