# Supplementary figures and images for: Uncovering the Novel Role of NR1D1 in Regulating BNIP3-Mediated Mitophagy in Ulcerative Colitis
Source: Int J Mol Sci. 2023 Sep 18;24(18):14222. doi: 10.3390/ijms241814222 (PMC10531686; doi:10.3390/ijms241814222)

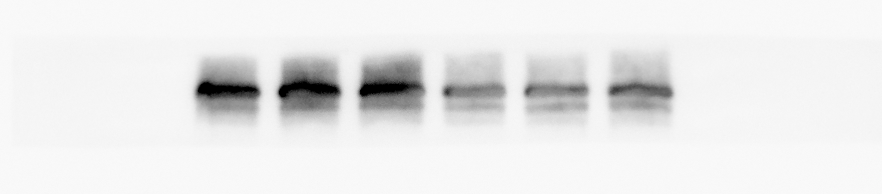

Supplement: Supplementary file 1 [file ijms-24-14222-s001.zip › ijms-2575368 supplementary/S2/BNIP3.png]

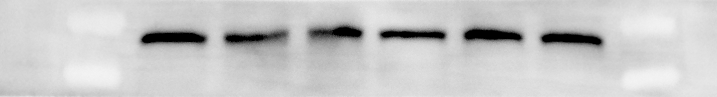

Supplement: Supplementary file 1 [file ijms-24-14222-s001.zip › ijms-2575368 supplementary/S2/GAPDH.png]

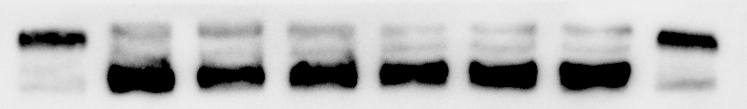

Supplement: Supplementary file 1 [file ijms-24-14222-s001.zip › ijms-2575368 supplementary/S2/NR1D1.png]

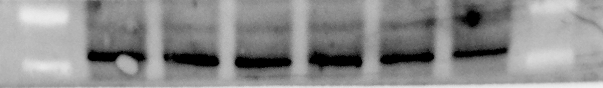

Supplement: Supplementary file 1 [file ijms-24-14222-s001.zip › ijms-2575368 supplementary/S3/BNIP3.png]

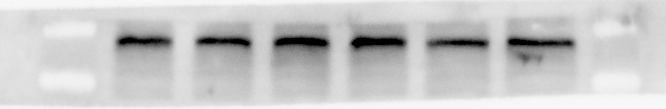

Supplement: Supplementary file 1 [file ijms-24-14222-s001.zip › ijms-2575368 supplementary/S3/GAPDH.png]

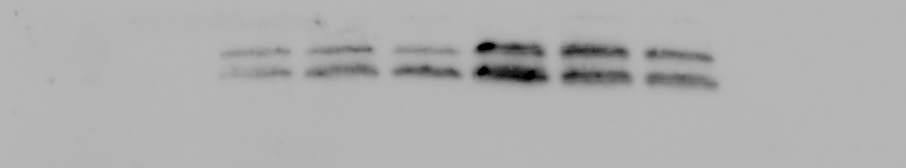

Supplement: Supplementary file 1 [file ijms-24-14222-s001.zip › ijms-2575368 supplementary/S3/LC3B.png]

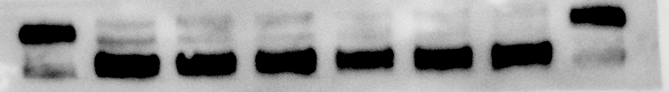

Supplement: Supplementary file 1 [file ijms-24-14222-s001.zip › ijms-2575368 supplementary/S3/NR1D1.png]

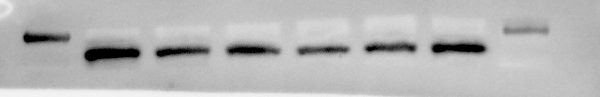

Supplement: Supplementary file 1 [file ijms-24-14222-s001.zip › ijms-2575368 supplementary/S4/GAPDH.png]

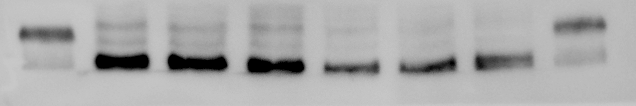

Supplement: Supplementary file 1 [file ijms-24-14222-s001.zip › ijms-2575368 supplementary/S4/NR1D1.png]
